# Modified prioritized DDPG algorithm for joint beamforming and RIS phase optimization in MISO downlink systems

**DOI:** 10.1038/s41598-026-36179-w

**Published:** 2026-02-11

**Authors:** Suzan Shukry, Yasmine Fahmy

**Affiliations:** 1 Electronics and Communications engineering Department, Misr University for Science and Technology (MUST), Giza, Egypt; 2 Higher Technological Institute, 6th of October city, postal code 4 Giza, Egypt; 3 Faculty of Engineering, Cairo University, Cairo, Egypt; 4 Wireless Intelligent Networks Center (WINC), Nile University, Giza, Egypt

**Keywords:** Engineering, Mathematics and computing

## Abstract

Reconfigurable Intelligent Surfaces (RIS) are emerging technology to enhance the 6G wireless communication systems by intelligently reconfiguring the propagation environment. This paper propose a Modified Prioritized Deep Deterministic Policy Gradient (MP-DDPG) algorithm for jointly optimizing beamforming at the Base Station (BS) and RIS phases in a Multiple Input Single Output (MISO) downlink communication system. The primary objective is to minimize transmitted powers while adhering to critical constraints such as maximum power limits and Quality of Service (QoS) requirements of the User’s Equipment (UE). The simulation results show that the MP-DDPG algorithm offers a robust and adaptive framework for addressing the inherent non-convexity and dynamic nature of such an optimization problem. Key findings highlight the remarkable resilience to imperfect Channel State Information (CSI), and the crucial trade-offs between performance, computational complexity, and signaling overhead.

## Introduction

Some innovative applications, such as data-driven, instantaneous, ultra-massive, and ubiquitous wireless connectivity applications are expected to be supported by 6G-and beyond communications^1^.

A data-driven application is characterized by the real-time collection and analysis of large volume of network data, which are used as the basis for intelligent decision-making processes. Such applications enable advanced functionalities to improve the efficiency of wireless communication systems. Instantaneous applications refer to highly reliable services that require very-fast response times. Examples of instantaneous applications including autonomous driving, remote robotic control, which require high performance and safety. Ultra-massive connectivity, is driven by the rapid growth of IoT and smart industry. ultra-massive connectivity requires a very large numbers of heterogeneous device connected, coordinated, and managed efficiently.

These applications require high data rates. High reliability and low latency. Innovative technologies are needed to support such high QoS requirements with lower cost, energy consumption, and hardware complexity^2,3^.

Reconfigurable Intelligent Surfaces (RISs), also known as Intelligent Reflecting Surfaces (IRSs), are one of the most promising technologies to enable 6G and beyond wireless communications. RISs are envisioned to enhance the spectrum and energy efficiency of wireless systems by reconfiguring the wireless propagation environment. Unlike traditional active components like relays, RISs are typically composed of numerous passive, low-power reflecting elements^4^. These elements can precisely adjust the phase and amplitude of incident electromagnetic waves, thereby re-directing signals towards desired receivers or mitigating detrimental interference^5,6^. By precisely controlling the reflection coefficients of its elements, an RIS can steer electromagnetic waves towards intended receivers, effectively mitigating signal propagation impairments, overcoming blockages, and transforming otherwise unfavorable propagation paths into beneficial ones^6,7^. The core functionality of an RIS is governed by the configurable phase shifts of its elements, which must be jointly optimized with the BS’s beamforming weights to maximize the received signal power.^8,9^. This capability allows RIS to maximize the total number of served devices, improve the signal-to-noise ratio (SNR) value, and increase the network sum rate, furthermore, RIS can contribute to increasing the covered area and enhancing energy collection capacity in hybrid communication-and-energy-harvesting scenarios^6^.

During the past years, many works have studied RIS-assisted wireless communication. In Ref.^10^, the authors investigate the characteristic of the downlink sum rate of an RIS-assisted wireless communication. The authors of Ref.^11^ study the achievable rate in a downlink RIS system. The authors of Ref.^12^ propose a low-complexity algorithm to maximize the weighted sum-rate of all users in a multiuser multiple-input single-output (MISO) downlink RIS-aided communication system; they also validate the proposed algorithm by numerical results.

The authors of Ref.^13^ consider RIS assisted (mmWave) non-orthogonal multiple access (NOMA) communication system. In their work users are partitioned into clusters of users. Different users’ clusters are served by subsets of RIS. To maximize the sum achievable rate, the authors propose a machine learning (ML) based three-stage algorithm that jointly optimizes user clustering, RIS partitioning, active beamforming, passive beamforming, and power allocation, taking into consideration the quality-of-service (QoS) requirements of each user and the maximum transmit power constraint of the base station (BS). Simulation results show that the proposed algorithm improves system throughput and achieve higher sum rates than conventional optimization algorithm.

Due to the growing demands for next-generation wireless networks, CELL-FREE (CF) system is proposed. In CF system to reduce the communication distance, there are a large number of access points (Aps) are distributed over the large service area. Reducing the communication distance between Aps reduces the shadow fading negative effects. The authors of Refs.^14,15^ consider RIS-aided cell free (CF) system. To maximizing the weighted sum-rate of all users the authors of Ref.^14^ propose a distributed beamforming algorithm. The proposed algorithm designs active and passive beamforming with the constraints of transmit power of Aps, and unit-modulus of RIS elements. Numerical results demonstrates that the proposed algorithm improves the performance compared with conventional local beamforming methods. To maximize the worst-case sum rate of the CF system the authors of Ref.^15^ propose a new algorithm. The proposed algorithm divides the problem into two subproblems. The subproblems are the RIS phase shifts optimization subproblem, and a precoding optimization subproblem. Numerical results shows that the proposed algorithm improves the performance with low complexity.

To maximize the user-achievable rate in Ref.^16^, the authors propose a new two-phase algorithm to optimize beamforming at the BS and the phase shifts at the RIS. The proposed algorithm is based on genetic algorithms. They used statistical channel information to reduce channel estimation overheads.

The work in Ref.^17^ represents a derivation of an analytical closed-form expression for the effective rate, taking into consideration some impairments such as location uncertainty and imperfect phase estimation. A Monte Carlo simulation validates the results and shows high accuracy.

The authors of Ref.^18^ propose a new protocol to maximize the achievable sum rate for both the single-user and multiuser cases. To overcome the difficulty of obtaining instantaneous CSI in RIS-assisted wireless communication systems, they propose a two-timescale transmission protocol. The authors study the effect of channel correlations in the BS-RIS and RIS-user links on the performance of the proposed algorithm for different values of SNR.

In Ref.^19^, the authors study the impact of CSI errors on the outage performance in RIS-aided wireless systems. They propose two algorithms, considering both multiuser and single-user cases. The simulation results show that the proposed algorithm significantly reduces the transmitted power at the BS with guaranteed outage performance.

Research efforts in RIS-assisted systems frequently focus on transmit power minimization as a key optimization area, as this directly contributes to reducing operational costs and environmental impact^8,9^. In the context of the transmit power optimization, the authors of Ref.^20^ develop an iterative mean-square error minimization approach in an RIS-aided MIMO network. The authors investigate the bit-per-Joule Energy Efficiency (EE) which is defined as the ratio of the information rate to the total power consumption and the Spectral Efficiency (SE) tradeoff.

Some studies aim to minimize transmit power while satisfying a minimum SNR at the receiver, which is a direct QoS constraint^9^. In Ref.^21^, the authors consider the case of a single RIS-sided wireless communication system, They study how to maximize the energy efficiency of a multi-user multiple-input single-output (MISO) system by optimizing the transmitted power at the BS and the phase shifts of the RIS, taking into consideration the constraints of maximum power and minimum QoS requirements. To formulate the optimization problem a realistic RIS power consumption model is developed.

In Ref.^22^, the authors study the resource allocation problem for a distributed RIS-aided wireless communication system where a number of RIS are spatially distributed. The authors consider the optimization problem of maximizing the energy efficiency for the single- and multiple-user scenarios and propose two suboptimal iterative algorithms with low complexity.

The authors of Ref.^23^ consider secure transmission for RIS-based downlink multiuser MISO systems. The authors study the limitations of acquiring CSI and to solve the optimization problem of minimizing the transmit power with the minimum SNR constraints, they propose particle swarm optimisation based technique.

Some Deep Reinforcement Learning (DRL) approaches might prioritize maximizing the weighted sum rate, they inherently treat transmit power as an optimization variable, subject to maximum power constraints, implying that efficient power usage is a core component of their design^24^. The authors of Ref.^25^ study the power minimization problem under the constraints of SNR requirements and RIS power budget. They present an optimisation-driven DDPG approach, which is a variation of DRL-based algorithms. In the paper, the DDPG algorithm generates only one part of the action, which is the passive beamforming. The active beamforming is generated using a model-based convex approximation optimization.

This paper introduces a DRL solution for optimizing beamforming and phase shifts in RIS-assisted MISO wireless systems. The solution targets the downlink of an outdoor cellular network where a multi-antenna BS, communicates with single-antenna mobile users through a direct link and a RIS. A key advantage of this proposal is that it does not require CSI acquisition, which is typically a complex challenge in RIS-assisted systems. The main objective is to minimize the power consumed by the BS for data transmission, aiming for energy-efficient wireless communication. This power minimization is constrained by a maximum transmit power at the BS and minimum QoS requirements at the User’s Equipment (UE), specifically in terms of Signal-to-Interference-Noise Ratio (SINR). The key contributions of this work are outlined as follows:

1. We propose the modification of the prioritized experience replay mechanism within DDPG. In our MP-DDPG, we introduce a novel priority metric that combines the distance in time between sample and its *K* nearest neighbor (KNN samples) and the reward of the sample.
2. Since conventional DDPG suffers from slow convergence and high-power consumption due to inefficient learning from past experiences, our MP-DDPG directly improves sample efficiency and convergence stability. This improvement leads to the faster convergence and superior performance as demonstrated in our results.
3. We study the performance in terms of total transmitted power, scalability, and the convergence speed. The simulation results show that our proposed MP-DDPG algorithm is more effective and practical for larger-scale systems compared to conventional DDPG.

In the following section, a literature review is presented for the DRL-based algorithms, their core principles and how they are used in the context of RIS, especially in the power minimization problem. In Sections 3 and 4, the system model under consideration and the problem formulation are detailed; then Section 5 is dedicated to the proposed deep reinforcement learning solution (MP-DDPG). Section 6 presents the simulation environment and results, while Section 7 discusses the complexity analysis. Finally, Section 8 concludes the paper.

## Literature review

A consistent observation across the literature is that joint optimization problems involving RIS and beamforming, especially with QoS and power constraints, are inherently non-convex and often classified as NP-hard, this poses a significant challenge for traditional optimization methods, leading to extremely high computational complexity. Deep Reinforcement Learning (DRL) algorithms ability to handle such complex, non-linear, and non-convex problems without relying on explicit mathematical models makes it a highly viable, and often superior, approach for achieving real-time optimization in dynamic wireless scenarios^26^

DRL represents a powerful paradigm in artificial intelligence that integrates deep neural networks with reinforcement learning, allowing DRL agents to learn optimal policies through interaction with complex, dynamic environments and receive feedback in the form of rewards or penalties, ultimately maximizing long-term cumulative returns^27,28^. Neural networks in DRL act as robust function approximators, enabling efficient processing of high-dimensional state spaces and discerning intricate relationships in raw input data^27^.

DRL’s model-free nature is crucial for its application in dynamic wireless environments. Unlike traditional model-based optimization techniques that struggle with unknown channel models and rapidly changing network conditions^29,30^, DRL can learn about radio channels without requiring explicit prior knowledge of the channel model or mobility patterns^6,31,32^. It achieves this by autonomously observing rewards and iteratively finding solutions to optimization problems^6^, directly addressing the limitations of traditional approaches and leading to robust performance and real-time adaptability in unpredictable wireless scenarios^29,32^. Furthermore, DRL algorithms have demonstrated significant efficiency in resolving the complexity of non-convex optimization problems in wireless communication systems^33^.

Developing a DRL solution for jointly optimizing BS beamforming and RIS phase shifts requires careful design of the Markov Decision Process (MDP) components: the **state space**, **action space**, and **reward function**.

The **state space** provides the DRL agent with comprehensive information about the wireless environment at each time step. For example, in sum-rate maximization problems, the state might include the current rate of each UE and cascaded channel information from each BS^26^. In more complex full-duplex systems, the state can encompass SINRs at both the BS and downlink UE from the previous time step, previously predicted RIS phase shifts and beamforming vectors, and the transmit powers of the BS and uplink UE^24^. For delay optimization problems, the state can be augmented with factors like the number of backlogged packets in buffers and current packet arrival rates^34^.

The **action space** defines all possible decisions the DRL agent can make, including the BS’s active beamforming vector and the RIS’s passive phase shifts^9^. Given the continuous nature of these variables, actions can be defined as optimal variables encompassing azimuth and elevation angles for beamforming, along with parameters for RIS and UEs, which may include both continuous angles and discrete association decisions components^26^. In some DRL frameworks, the actor network directly outputs predicted RIS phase shifts, the real and imaginary parts of transmit and receive beamforming vectors, and transmit powers. These outputs are often normalized (using $\tanh$ activation) and scaled to their valid ranges ($[0, 2\pi ]$ for RIS phases, unit norm for beamformers)^24^. To facilitate exploration and accelerate convergence during training, Gaussian noise is often added to the action space. For practical implementation and to reduce signaling overhead, the framework can be extended to handle quantized RIS phase shifts, where the neural network might predict probabilities for selecting specific quantized phase values, or passive elements can be grouped^24^.

The **reward function** is crucial for guiding the DRL agent’s learning by providing immediate feedback on its actions. For power minimization under QoS constraints, the reward function must incentivize lower transmit power while penalizing QoS violations. In scenarios focused on maximizing sum-rate, the reward function is often directly equivalent to the objective equation, such as the sum of logarithmic rates^26^. For maximizing a weighted sum rate, the reward can be calculated as a weighted sum of uplink and downlink data rates, with weights balancing different traffic types^24^. This type of reward implicitly incorporates channel CSI, guiding the agent towards satisfying underlying QoS requirements related to signal quality.

Table 1 provides an overview of prominent DRL model-free algorithms and their typical applications within the context of RIS-aided wireless systems. Various DRL algorithms adapted for RIS-based wireless communication systems include Deep Q-Network (DQN), Deep Deterministic Policy Gradient (DDPG), Proximal Policy Optimization (PPO), and Soft Actor-Critic (SAC) each offering distinct advantages depending on the characteristics of the problem. DQN is foundational for discrete action spaces^6,28^. However, the continuous nature of beamforming weights and RIS phase shifts often necessitates algorithms capable of handling continuous action spaces, leading to the use of DDPG, PPO, and SAC^6,26,36^. DDPG is effective for dynamic beamforming design involving continuous state and action spaces due to its double-network architecture^24,36^. PPO is a robust model-free, on-policy, actor-critic method often used for optimizing gathered energy and sum rate^33^. SAC DRL is frequently proposed for complex joint optimization problems, such as joint beamforming and BS-RIS-UE association in mmWave networks, particularly for intractable non-convex problems with continuous action spaces, by incorporating a maximum entropy framework in the reward function^26^. The newer algorithms are more sophisticated and can be precisely tailored to allow for more detailed control and better overall performance.

**Table 1:** Overview of DRL Algorithms for RIS-Aided Wireless Systems.

**Algorithm**	**Space**	**Learning**	**Primary Objectives**	**Strengths/Notes**
**DQN**^27,33^	Discrete	Off-Policy	Sum-rate maximization, Data rate for V2I users	Good for discrete problems; often combined with attention mechanisms.
**PPO**^33–35^	Continuous	On-Policy	Energy Efficiency, Sum-rate maximization, Average delay optimization, QoS assurance	Robust policy learning; balances performance and computational complexity; nearly as efficient as exhaustive search.
**DDPG**^24,31,33,36^	Continuous	Off-Policy	Sum-rate maximization, Energy Efficiency, Weighted sum rate (UL/DL), Transmit power minimization	Handles high-dimensional continuous state/action spaces; actor-critic architecture; can cope with channel uncertainty.
**SAC**^26^	Continuous	Off-Policy	Sum-rate maximization, Joint beamforming and association	Incorporates maximum entropy for robust exploration and avoiding local optima.
**TD3**^37^	Continuous	Off-Policy	Sum-rate maximization	Twin Delayed DDPG, addresses overestimation bias in DDPG.
**SD3**^33^	Continuous	Off-Policy	Energy Harvesting Efficiency	Used for performance enhancement, often compared with PPO.

Specifically, many papers suggested various DRL approaches for joint beamforming and RIS phase optimization, these papers are summarized in Table 2 highlighting their system models, objectives, and key features.

**Table 2:** DRL Approaches for Joint Beamforming and RIS Phase Optimization.

Ref.	Model	Algorithm	Objective	Constraints	Features
^9^	RIS-aided MU-MISO	DRL (Modified Vanilla)	Max Sum Downlink Rate	Phase-dependent reflection amplitude, Imperfect CSI, Hardware impairments	Outperforms vanilla DRL under mismatch; first DRL for phase-dependent amplitude model.
^26^	RIS-assisted Multi-BS Multi-UE MISO (mmWave)	SAC-DRL	Max Downlink Sum-Rate	Max Transmit Power (BS), Min QoS (UE Rate), BS-RIS-UE association	Addresses NP-hard non-convex problem; learns with less prior info; avoids local optima.
^24^	RIS-assisted Full-Duplex (FD) UL/DL	DDPG (MSF-DRL)	Max Weighted Sum Rate (UL/DL)	Max Transmit Power (BS/ULue), RIS phase magnitude = 1	CSI-oblivious (minimal signaling feedback); two-stage learning for SI cancellation; handles quantized/grouped phases.
^36^	Cell-free network (Uplink)	DDPG	Max Long-term Energy Efficiency (EE)	(Implicitly through EE maximization)	Dynamic beamforming with continuous state/action space; converges to optimal EE.
^33^	RIS-aided NOMA CRNs / UAV-RIS	DDPG, PPO, DDQN, SD3	Max Sum-Rate, Max EH Efficiency, Max Achievable Data Rate	Channel uncertainty, Limited battery capacity, QoS (latency/reliability), Power budget	Efficient for non-convex problems; DDPG for continuous spaces; PPO for robust policy; DDQN for discrete.
^34,35^	RIS-assisted OFDM (Downlink)	Hybrid DRL (PPO-$\Theta$, PPO-N)	Optimize Average Delay	(Implicitly through delay optimization)	Handles mixed action space; incorporates backlogged packets/arrivals in state; transfer learning for efficiency.

Various DRL algorithms, including DDPG, PPO, and SAC, have been successfully employed, showcasing their adaptability to continuous action spaces inherent in beamforming and phase shift control.

Specifically, there is a significant amount of research in the area of using DDPG algorithms to solve this problem. DDPG is a deep reinforcement learning algorithm that uses an actor-critic approach to learn deterministic policies in continuous action spaces. It involves two neural networks: an actor network that outputs actions and a critic network that evaluates the value of those actions. The DDPG is well-suited and widely researched solution for this problem due to the following factors:

- **Continuous Action Space**: The active beamforming vector at the BS and the passive phase shifts at the RIS are continuous variables. DDPG is specifically designed for environments with continuous action spaces, making it a natural fit for this type of optimization problem. This is highlighted in the literature, where DDPG is noted for its “effectiveness in dynamic beamforming design involving continuous state and action spaces”.
- **Joint Optimization Capability**: Numerous studies leverage DDPG to jointly optimize both the transmit beamforming at the BS and the RIS phase shifts. For instance,^24^ uses DDPG (specifically, MSF-DRL) to maximize weighted sum rates in RIS-assisted Full-Duplex systems, which involves optimizing transmit powers and RIS phase shifts.^6^ uses the Twin Delayed DDPG (TD3) method, a DDPG variant, for optimizing RIS phase shifts to minimize the transmission power in RIS-aided MISO-OFDM systems.
- **Handling Non-Convexity**: The problem you described (power minimization under QoS constraints with joint beamforming and RIS phases) is inherently non-convex and often NP-hard. DRL algorithms, including DDPG, are well-suited to handle such complex, non-linear, and non-convex problems without relying on explicit mathematical models, which makes them a viable and often superior approach for real-time optimization.
- **Addressing Practical Challenges**: DDPG-based solutions have also been shown to be robust to practical challenges such as imperfect CSI and hardware impairments, further increasing their applicability in real-world wireless systems. Studies discuss how DDPG can operate with minimal signaling feedback, which is crucial for practical deployments.

Traditional DDPG uses a random sampling of experiences for training, which can be inefficient. Prioritized experience replay focuses on sampling more important experiences (e.g., those with higher errors) to improve learning speed and sample utilization. Modifications to DDPG typically involve incorporating techniques like prioritized experience replay to improve efficiency and convergence, or using fractional-order learning to accelerate training. In this work, a Modified Prioritized Deep Deterministic Policy Gradient (MP-DDPG) which is an enhanced version of the standard DDPG algorithm, is explored to solve the problem under consideration.

## System model

**Fig. 1:**
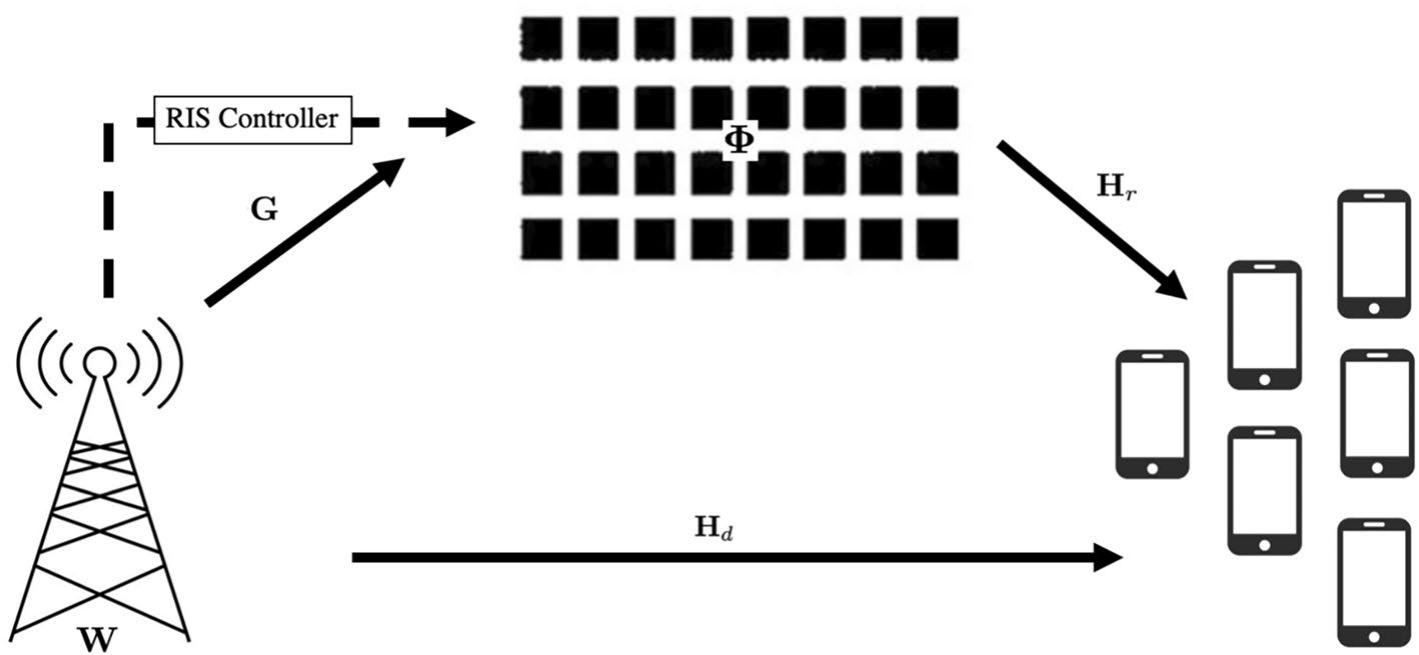
System Model.

The system under consideration,as shown in Fig. 1, involves a BS, in the context of 4G/5G mobile networks, referred to as an Evolved Node B (eNB), equipped with multiple transmit antennas ($N_t$ transmit antennas), serving *K* single-antenna UEs in the downlink. This configuration defines a Multiple users Multiple Input Single Output (MU-MISO) communication system. A Reconfigurable Intelligent Surface (RIS) is strategically deployed to assist this communication, particularly in scenarios where direct links between the BS and UE are weak or obstructed due to blockages^9,24^ The RIS functions as a passive reflector, intelligently altering the propagation path to enhance signal reception at the UE^9^.

The reflectors of the RIS are *M* passive elements so the RIS does not consume any transmit power. The amplification gain of the RIS comes from the adjustment of the reflecting elements’ phase^21^. For simplicity, we consider the maximum signal reflection, hence we set the amplitude reflection coefficient to unity.

Let $X \in \mathbb {C}^{K \times 1}$ represent the transmitted vector from eNB to the *K* UEs where every element $x_k$ is the transmitted data to the $k^{th}$ UE where $k=1,2, \cdots K$, and $\textbf{W} \in \mathbb {C}^{N_t \times K}$ denote the active transmit beamforming matrix applied at the BS. Let $\textbf{H}_d \in \mathbb {C}^{K \times N_t}$ represents the direct channel matrix from the eNB to the *K* UEs, while $\textbf{G} \in \mathbb {C}^{M \times N_t}$ represents the channel matrix from the eNB to the RIS.

The RIS introduces phase shifts to incident signals. This is represented by a diagonal matrix ${{\Phi }} \in \mathbb {C}^{M \times M}$, where ${{\Phi }} = \text {diag}(\phi _1, \phi _2, \dots , \phi _M)$. Each element $\phi _m$ corresponds to the phase shift induced by the $m-{th}$ reflecting element. For passive RIS elements, the magnitude of each phase shift is unity, typically expressed as $\phi _m = e^{j\theta _m}$, where $\theta _m \in [0, 2\pi )$ is the phase shift angle. Let the $\textbf{H}_r \in \mathbb {C}^{K \times M}$ represents the channel vector from the RIS to the *K* UEs. Finally, let *N* be the additive white Gaussian noise (AWGN) vector at the *K* UEs receivers, where each element $n_k \sim \mathcal{C}\mathcal{N}(0, \sigma ^2)$.

The Received Signal vector at the UEs, $Y \in \mathbb {C}^{K \times 1}$ is the superposition of the signal traveling through the direct path and the signal reflected by the RIS and can be given as

1 $$\begin{aligned} Y = (\textbf{H}_d^H + \textbf{H}_r^H {{\Phi }} \textbf{G}) \textbf{W} X + N \end{aligned}$$

The SINR, $\gamma _{k}(\Phi , W)$, at the $k^{th}$ UE is given by:

2 $$\begin{aligned} \gamma _{k}({{\Phi }}, \textbf{W}) = \frac{\vert ( h_{kd} + h_{kr} {{\Phi }} \textbf{G})\textbf{W} X\vert ^2}{\sum _{i=1,i\ne k}^K \vert (h_{id}+h_{ir} {{\Phi }} \textbf{G})\textbf{W} X\vert ^2 + \sigma ^2} \end{aligned}$$

where $h_{kd}$ is the $k^{th}$ row of the matrix $\mathbf {H_{d}}$ and $h_{kr}$ is the $k^{th}$ row of the matrix $\mathbf {H_{r}}$ corresponding to the UE *k*.

## Optimization problem formulation

The core problem addressed in this context is formulated as a nonconvex optimization challenge. The objective is to minimize the total transmit power at the eNB by jointly optimizing the active beamforming vector at the eNB and the passive phase shifts at the RIS. This minimization must adhere to crucial constraints, including a maximum allowable transmit power at the eNB and minimum QoS requirements for the User Equipment. This problem is inherently nonconvex.

### Objective function

The core objective is to jointly optimize the active beamforming matrix $\textbf{W}$ at the eNB and the passive phase shifts ${{\Phi }}$ at the RIS to minimize the total transmitted power, which is represented by the the squared Frobenius norm of the beamforming vector $\textbf{W}$ (i.e. $\min _{\textbf{W}, {{\Phi }}} \vert |\textbf{W} \Vert ^2$)

### Constraints

To ensure practical feasibility and user satisfaction, the power minimization objective must adhere to specific constraints. The maximum transmit power constraint limits the power consumption of the eNB that must not exceed a predefined maximum allowable power, $\vert |\textbf{W}|\vert ^2 \le P_{\max }$, preventing excessive interference to other users and respecting hardware limitations^24^. The QoS constraint guarantees a minimum level of service for the UE, typically expressed as a minimum achievable data rate or SINR^8,9,26^. For example, a minimum communication rate for each UE is a common QoS requirement, $\gamma _{k}({{\Phi }}, \textbf{W}) \ge \gamma _{\min }, \quad \text {for } k = 1, \dots , K$^26^. Additionally, the constant-modulus constraint for RIS phase shifts, where the magnitude of each element is constrained to 1, is another fundamental physical limitation that must be satisfied, $\vert \phi _m\vert = 1, \text {for } m = 1, \dots , M$^8,24^.

Thus, the optimization problem can be formulated as follows:

3a $$\begin{aligned} & &\min _{\textbf{W}, {{\Phi }}} \vert |\textbf{W} \Vert ^2 \end{aligned}$$

3b $$\begin{aligned}&\text {s.t.} & \gamma _{k}({{\Phi }}, \textbf{W}) \ge \gamma _{\min },&\text {for } k = 1, \dots , K \end{aligned}$$

3c $$\begin{aligned} & &\vert \mathbf |{W}|\vert ^2 \le P_{\max } \end{aligned}$$

3d $$\begin{aligned} & &\vert \phi _m\vert = 1,&\text {for } m = 1, \dots , M \end{aligned}$$

## Proposed MP-DDPG algorithm

To solve the non-convex optimization problem in (3a), we propose the use of DRL which combines Reinforcement Learning (RL) and Deep Neural Network (DNN). RL is used to solve Markov Decision Problem (MDP) where the agent decides to take actions based on what it learns. DRL can be used to solve optimization problems when the states of MDP are of very high dimension. In our work, we propose to use a modifier prioritized Deep Deterministic policy Gradient (MP-DDPG). The traditional DDPG is a model free deep reinforcement learning algorithm which is effective in solving high dimension continuous optimization problems. DDPG is an actor-critic RL algorithm. In DDPG agent interact with the environment to generate experience data. Experience data is stored in finite size memory called experience replay buffer. Each iteration, data in experience replay buffer are sampled randomly to update the network. A fixed size of samples is randomly selected. Experience replay removes the correlation between observations samples which may result in instability in reinforcement learning process. Other limitations of DDPG is occurrence of local minimum. To increase the convergence speed and reduce the limitations in DDPG method, we propose an enhanced algorithm.

Conventional DDPG is actor-critics framework in which the workflow can be described as follow: First, the agent interacts with the environment to collect data and store it in experience replay buffer. Secondly, the sampled data from the replay buffer is used to update the actor- critics networks.

The basic components of a single agent DDPG learning architecture are as follows:

- **State Space** *S* **:** In our work, the action space consists of the eNB beamform vector, and RIS elements phase shift. The system status which is represented by the state space consists of the previous phases of all the RIS elements, previous beamforming ,received power, and the received SINR. For each learning iteration *t*, we define the state vector $S_t$ that represents a vector of the previous phases of all the RIS elements ($\Phi$), the previous active beamforming at eNB (*W*), received power, and SINR values.
- **Action Space** *A* **:** for each learning iteration *t*, the action vector $a_t$ represents the continues change in the RIS element phases for each element, and eNB active beamforming. The action determines the next state $S_{t+1}$.
- **Reward:** we consider Weighted Multi-Objective Reward where
  4 $$\begin{aligned} r(t)= - \left( \zeta _1 \frac{P_t}{P_{max}}+ \zeta _2 \gamma _c + \zeta _3 P_c \right) \end{aligned}$$
  where $\gamma _c$ is an indication of the satisfaction of the SINR constraint of (3b), $\gamma _c =1$ if the constraint is satisfied, and $\gamma _c = 0$ otherwisee, $P_c$ is an indication of the satisfaction of the maximum power constraint of (3c), where $P_c =1$ if the constraint is satisfied and $P_c = 0$ otherwise, $P_t$ is the transmitted power, finally $\zeta _1$, $\zeta _2$ and $\zeta _3$ are the adjustable weights. In our work, we assume equal weights ($\zeta _1= \zeta _2 = \zeta _3 = 1$).

DDPG incorporates two deep neural networks, single actor network, and single critic network. The input to the actor network is the state vector. For each state $s_t \in S$, the actor generates the continues action $a_t \in A$ which is optimized for the environment applying the policy $\pi$. The action $a_t$ is generated based on the policy of the actor $\pi$, and the current state $s_t$

5 $$\begin{aligned} a_t=\pi (s_t | \theta _\pi )+N_t \end{aligned}$$

Where $N_t$ is the exploration noise that generated randomly, and $\theta _\pi$ is the parameters of the actor network. The critic takes the state vector and the action taken as inputs. For each state action pairs, the critic estimates the *Q* value. The target value $y_t$ is given by

6 $$\begin{aligned} y_t=r_{t+1}+\gamma Q' \left( s_{t+1},\pi ' (s_{t+1} | \theta ^{\pi '}) | \theta ^{Q'} \right) , \end{aligned}$$

where $r_{t+1}$ is the reward, $\gamma$ is the discount factor. $Q'$,and ${\pi '}$ are the target critic and actor networks with parameters $\theta ^{Q'}$ and $\theta ^{\pi '}$ respectively.

Let $L(\theta ^Q)$ be the loss function of the critic network. During network training, the network is updated by minimizing the loss function. If the batch size is *B*, then the loss function is given by

7 $$\begin{aligned} L(\theta ^Q)= \frac{1}{B} \sum _{i}(y_i-Q(s_i,a_i | \theta ^Q))^2 \end{aligned}$$

In Conventional DDPG, random selection of samples from replay buffer breaks the correlation between samples. To improve the convergence time, the prioritized replay buffer is applied^38^^39^.

In our work, we assign a weight for each sample in the replay buffer. The weight reflects the importance of the sample. The selection of samples from the replay buffer depends on the successful performance of the sample while training, which is reflected in the sample weight.

The selected samples from the replay buffer should be diverse in states over time, and at the same time could improve policy and value function.

Let us define the distance in time between the sample *i* and *j*, $d(t_i,t_j )=\Vert s_{ti}- s_{tj} \Vert$, which represents the Euclidean distance in state space between the samples. To reduce computation overhead, instead of getting the distance between each sample and all other samples in the replay buffer we get the distance between each sample and its *K* nearest neighbor (KNN samples). If the reward of sample *i* is $r_i$. then we can calculate the priority of sample *i* as

8 $$\begin{aligned} p_i=\frac{\vert r_i \vert }{\sum _{j=1}^{K} d(t_i,t_j)} \end{aligned}$$

**Algorithm 1:**
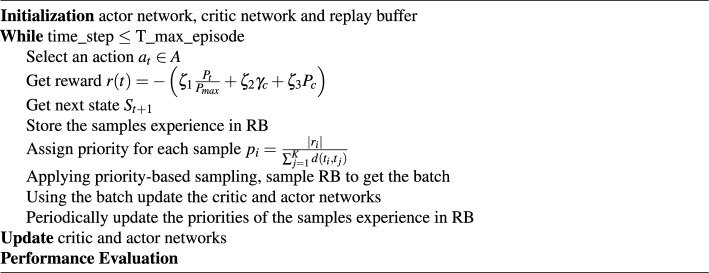
Modified Priority-DDPG.

## Simulation and results

In this section, we evaluate the performance of the proposed algorithm using MATLAB-based Monte Carlo simulations. We assume the channels are Rician fading with log-distance path loss^40–43^, consider one reflection only by the RIS and neglect any signals reflected more than once, we have:

$$\begin{aligned} G=G^{LOS} \sqrt{\frac{\beta _{AR}}{1+\beta _{AR}} } + G^{NLOS} \sqrt{\frac{1}{1+\beta _{AR}} } \end{aligned}$$

where $G^{LOS}$ and $G^{NLOS}$ denot the Line of Sight (LoS), and Non Line of Sight (LNoS) components respectively and $\beta _{AR}$ denotes the Rician factor. For modeling BS-UE channel and the RIS-UE channel, we use the same model. In our work we assume $\beta _{AR}$ =0, $\beta _{AU}=0$, and $\beta _{RU}=1$^42^.

**Fig. 2:**
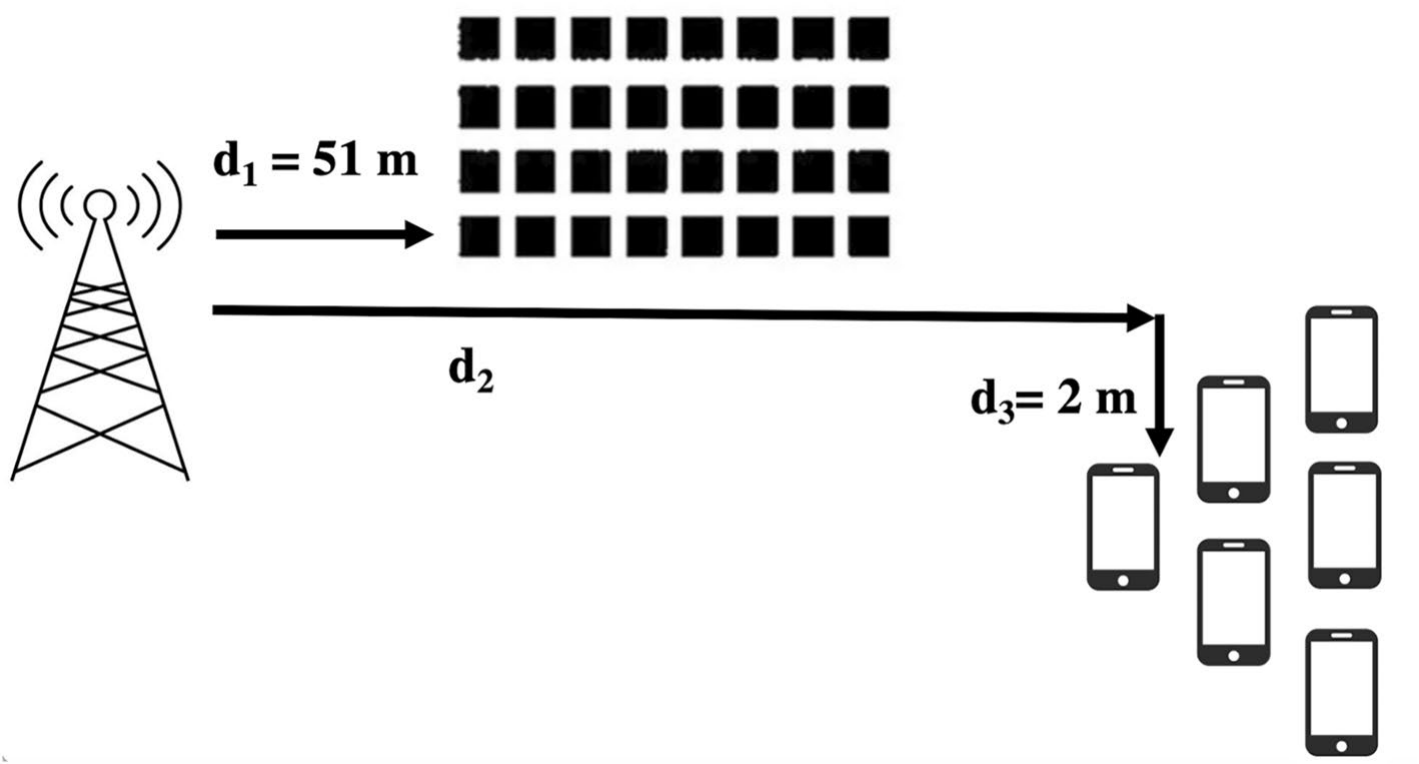
Simulation Scenario.

The distance dependent pathloss *PL*(*d*) is also taken into consideration. Our simulation scenario is shown in Fig. 2, where $d_1= 51 \ m$ and $d_3 =2 \ m$. The distance between the eNB and the UE is then given by $d_{BU}=\sqrt{d_2^2+d_3^2}$, the distance between the RIS and the UE is given by $d_{RU}=\sqrt{(d_2-d_1)^2+d_3^2}$.

We also consider the replay buffer of size 10000, while the batch size *B* is 100 samples. The neural networks architectures and hyperparameters of the MP-DDPG under consideration are summarized in Table 3.

**Table 3:** MP-DDPG Neural Network Architectures and Hyperparameters.

**Component**	**Parameter**	**Specification**
Actor Network	Input Layer	$M+ N_tK +2K$
	Hidden Layer 1	400 neurons
	Hidden Layer 2	300 neurons
	Output Layer	$M + N_t K$
Critic Network	Input Layer (State)	$2M + 2N_tK +2K$
	Hidden Layer 1	400 neurons
	Hidden Layer 2	300 neurons
	Output Layer	1 neuron, Activation, Linear
Training Hyperparameters	Critic Learning Rate	$10^{-3}$
	Actor Learning Rate	$10^{-4}$
	Discount Factor	0.99
	Soft Update Rate	0.001
	Replay Buffer Size	10000
	Batch Size (*B*)	100

To evaluate the performance of the proposed scheme, we compare the performance of the proposed MP-DDPG scheme with two well-known schemes. The first one is the Particle Swarm Optimization (PSO)^23^, and the second is Deep Deterministic Policy Gradient (DDPG)^24,33,36^. PSO is a canonical and well-understood classical optimization approach, while DDPG is a reinforcement learning-based technique, which is effective for continuous action space.

**Fig. 3:**
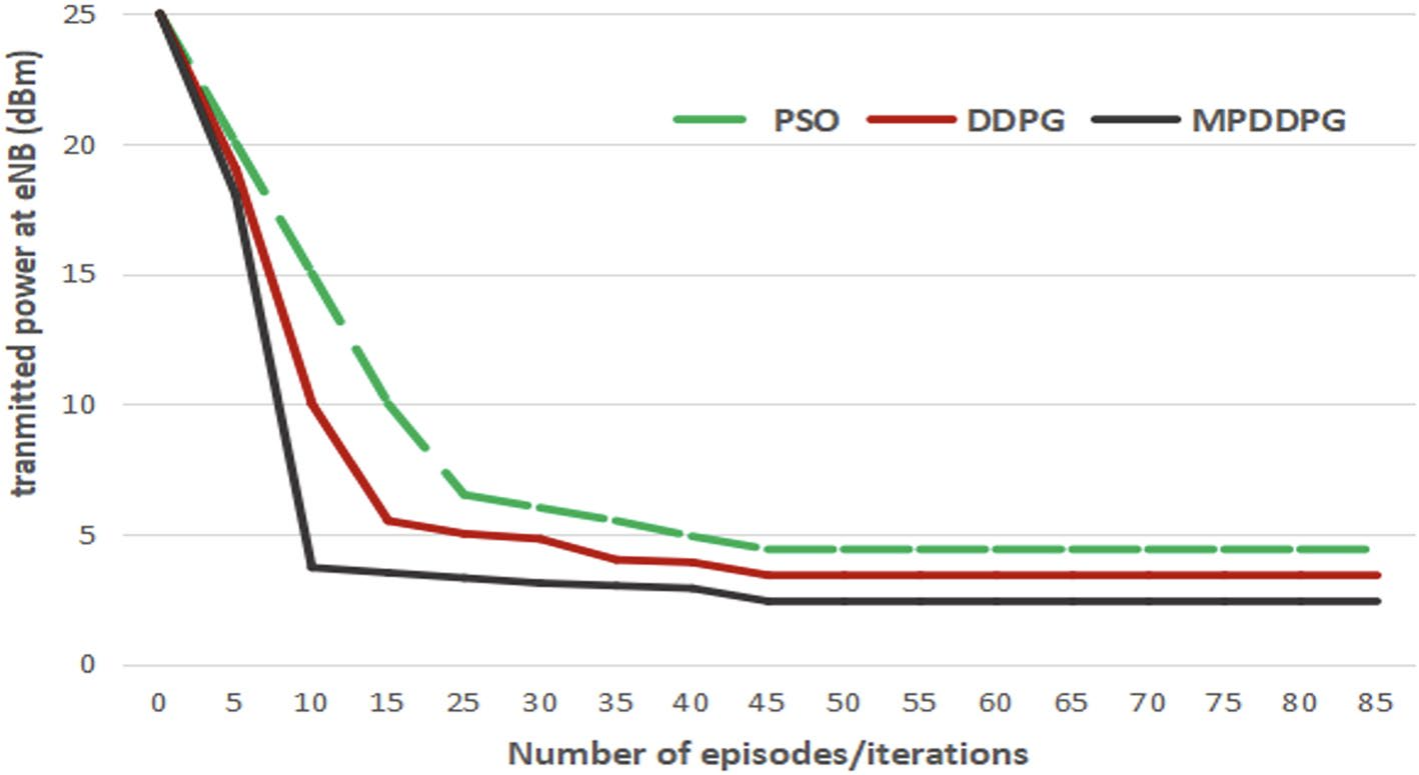
The transmitter power at eNB vs number of episodes/iterations.

Figure 3 investigates the transmitted power from eNB for different numbers of episodes/iterations. It compares the transmitted power for PSO, DDPG and MP-DDPG with the number of iterations /episodes.

Figure 3 demonstrates that applying DRL to solve the optimization problem improves the performance in terms of reducing the transmitted power from BS. The convergence of the optimization algorithm is very critical. It is clear from the figure that DRL (DDPG, and MP-DDPG) converges to the minimum transmitted power faster than PSO. The performance of DDPG is further improved when applying priority RB. The improvement results from a better training process due to the selection of good sample sets for training.

**Fig. 4:**
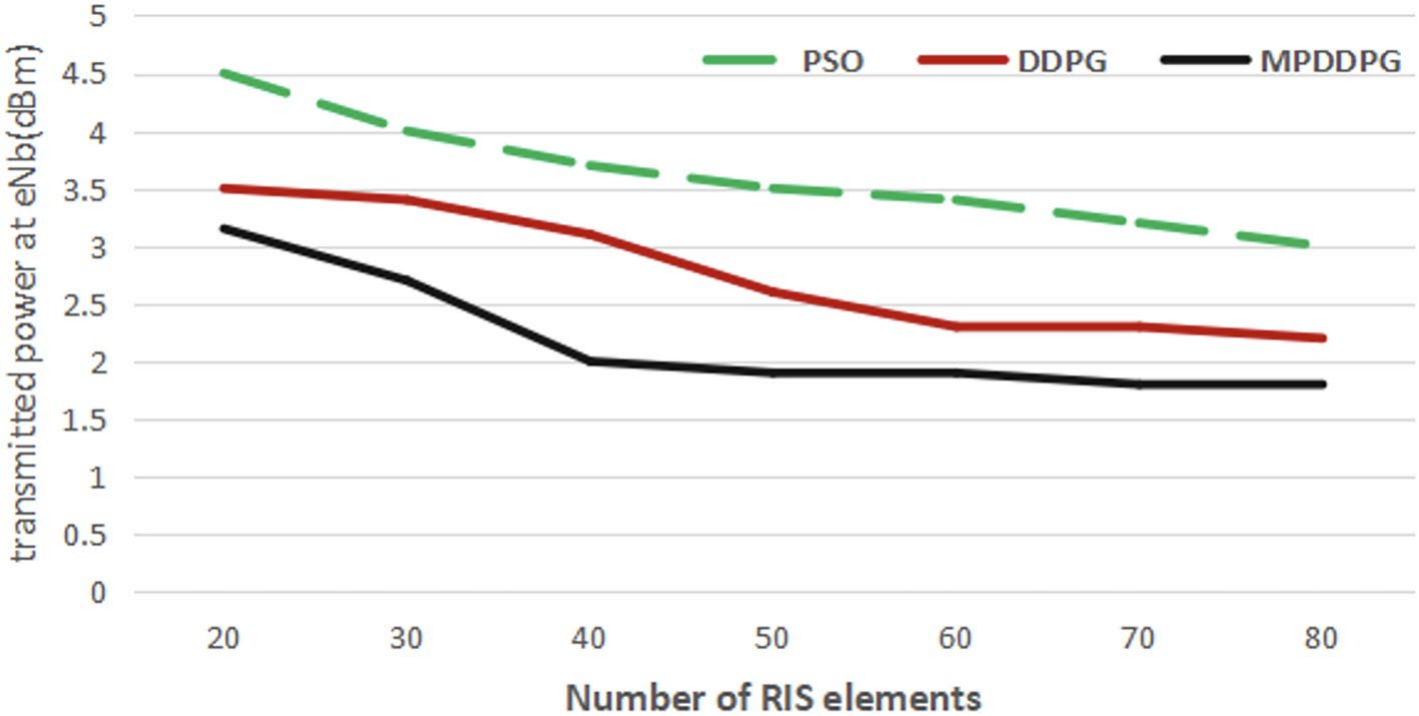
The transmitter power at eNB vs number of RIS elements.

Figure 4 investigates the effect of increasing the number of RIS elements on the transmission power in BS. The figure shows the transmitted power at eNB for different numbers of RIS elements. The figure also shows that DRL based solution reach the minimum for a smaller number of RIS elements than the PSO. It is also clear that the proposed MP-DDPG algorithm reaches to the minimum transmitted power for a smaller number of RIS elements that DDPG. Applying the proposed MP-DDPG algorithm and increasing the number of RIS elements from 20 to 30 elements reduces the transmitted power by $35\%$, while achieving the same percentage of improvement in performance (same reduction of transmitted power) the number of RIS elements must increases from 20 to 40 elements when applying DDPG and from 20 to 80 elements when applying PSO.

**Fig. 5:**
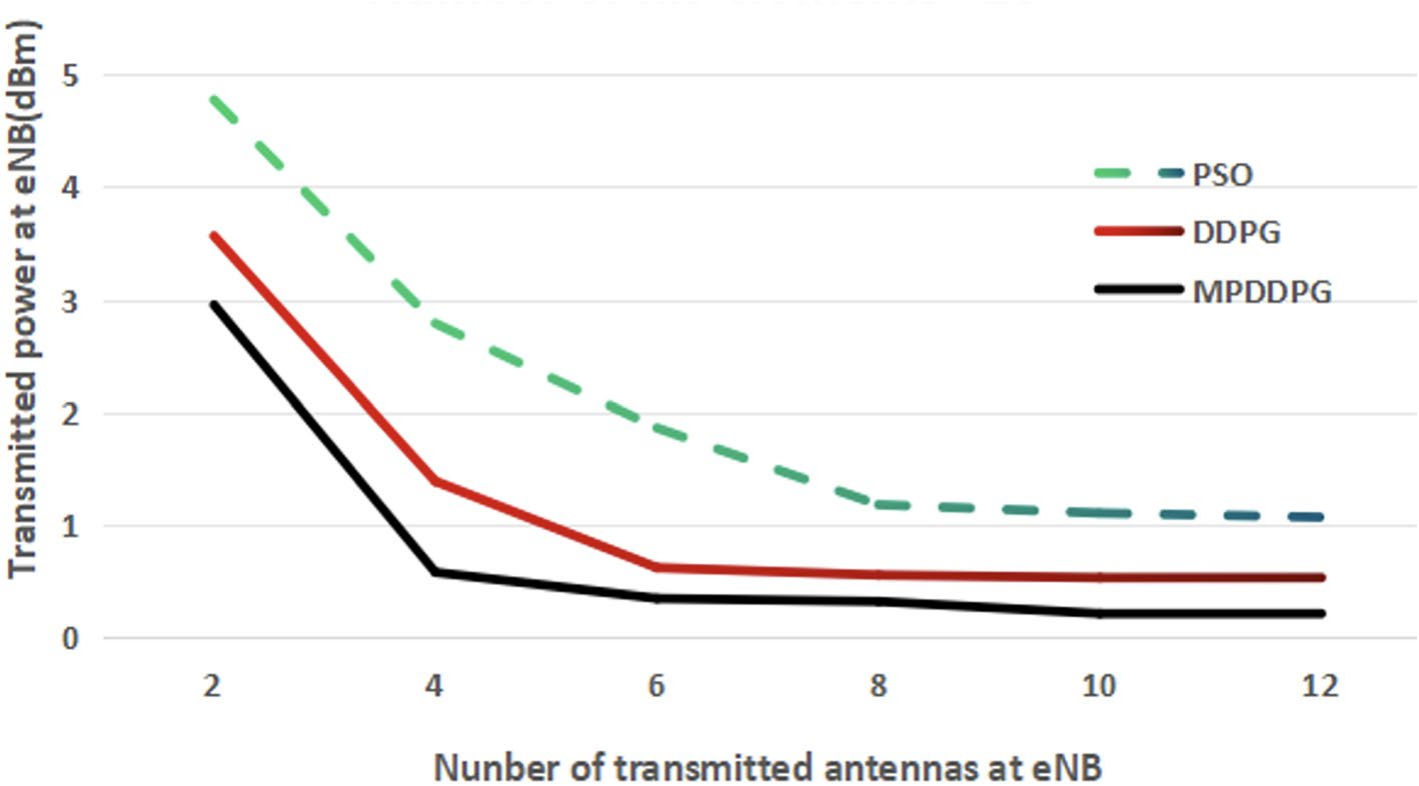
The transmitted power at eNB versus the number of antennas at eNB (Number of RIS elements =20).

Figure 5 illustrates the transmitted power at eNB with the increasing number of antennas at BS. It is shown in the figure that increasing the number of antennas at eNB improves the performance for all algorithm due to increasing the special diversity. When applying the DDPG and MPDDG, the transmitted power is saturated at certain minimum value at certain number of antennas at eNB. MP-DDPG results in lower minimum saturated value of transmitted power at a smaller number of eNB antennas.

Form Figs. 4 and 5, we can see that applying MP-DDPG algorithm results in improving the performance with less increasing in system complexity in terms of number of RIS elements, and number of eNB antennas.

From Figs. 3, 4 and 5, it is clear that applying MP-DDPG improves the performance than applying DDPG. This improves comes from the selection of most effective samples in the training of the DRL model instead of selecting them randomly.

## Complexity analysis

In the conventional DDPG algorithm, the computational complexity per update iteration is dominated by the forward and backward propagation through the actor and critic networks. If each network has *L* layers with *N* neurons per layer, the complexity is approximately $O(B.L.N^2)$ where *B* is the batch size used during training. Replay buffer sampling is random, with complexity *O*(*B*). Thus, the overall per-iteration complexity of the DDPG is $O(B.L.N^2)$

The proposed MP-DDPG algorithm introduces the following additional steps: Priority Assignment, Priority-Based Sampling, and then Neural Network Training. The total computational complexity of MP-DDPG arises from the complexity of all these stages. If the buffer size is *R*, *B* is the batch size, and *K* is the number of the number of nearest neighbors used for distance-based priority calculation, the complexity of the priority assignments for each sample based on the distance to KNN is *O*(*R*.*K*.*d*) where *d* is the state-space dimension.

For the Priority-Based Sampling, instead of the uniform random sampling, samples are drawn according to their assigned priority. Using a tree-based data structure, this operation has a complexity of $O(B.\log {R})$.

Finally, in the Neural Network Training step, the actor and critic networks updates remain identical to conventional DDPG, with complexity and it is of order $O(B.L.N^2)$.

The overall complexity of MP-DDPG is then of order $O(R.K.d) + O(B.\log {R})+ O(B.L.N^2)$.

Although MP-DDPG adds extra overhead due to to the priority assignment and structured sampling, this increase is justified by the significant improvement in performance. As indicated by Fig. 3, MP-DDPG converges to the optimal policy in significantly fewer episodes than DDPG. The prioritization mechanism ensures that each update is performed on a more informative and diverse set of experiences, leading to more efficient learning.

## Conclusion

This research proposes a Modified Prioritized Deep Deterministic Policy Gradient (MP-DDPG) algorithm for robust beamforming optimization in Reconfigurable Intelligent Surface (RIS)-assisted wireless communication systems. The primary objective of this algorithm is to minimize transmitted power at the eNB by jointly optimizing beamforming at the eNB and the phase shifts of the RIS, while satisfying constraints such as maximum transmit power and QoS requirements of the User Equipment (UE). A key advantage of the proposed DRL solution is its ability to operate without requiring instantaneous CSI acquisition, which is typically complex in RIS-assisted systems. The simulation results consistently demonstrate the superior performance of the MP-DDPG algorithm. Compared to Particle Swarm Optimization (PSO) and conventional DDPG, the MP-DDPG algorithm achieves faster convergence to the minimum transmitted power. Furthermore, it attains a lower minimum saturated transmitted power with a smaller number of RIS elements and eNB antennas, indicating improved performance with reduced system complexity. This performance improvement is attributed to the prioritized experience replay mechanism within MP-DDPG, which selects more effective samples to train the DRL model instead of random selection, leading to a better training process. The findings highlight the potential of MP-DDPG as an efficient and robust solution to optimize resource allocation in future 6G and beyond wireless communication networks.

## Data Availability

The simulated data, used in this study, was generated using Matlab code which can be provided from the corresponding author upon reasonable request.
